# Feeding Arsenic-Containing Rice Bran to Growing Pigs: Growth Performance, Arsenic Tissue Distribution, and Arsenic Excretion

**DOI:** 10.3390/ijerph17228530

**Published:** 2020-11-17

**Authors:** Shengfa F. Liao, M. Shamimul Hasan, Zhongyue Yang, Andrew W. Stevens, James Brett, Zhaohua Peng

**Affiliations:** 1 Department of Animal and Dairy Sciences, Mississippi State University, Mississippi State, MS 39762, USA; msh435@msstate.edu (M.S.H.); yzyang@ucdavis.edu (Z.Y.); 2 Department of Agricultural and Applied Economics, University of Wisconsin–Madison, Madison, WI 53706, USA; awstevens@wisc.edu; 3 Department of Veterinary Pathobiology and Population Medicine, Mississippi State University, Mississippi State, MS 39762, USA; jbrett@cvm.msstate.edu; 4 Department of Biochemistry, Molecular Biology, Entomology, and Plant Pathology, Mississippi State University, Mississippi State, MS 39762, USA

**Keywords:** arsenic, rice bran, pig, tissue distribution, excretion, growth performance

## Abstract

This research was conducted to study the growth performance, arsenic (As) tissue distribution, and As excretion of pigs fed As-containing rice bran. Twenty gilts (26.3 kg) were randomly assigned to 3 dietary treatments (*n* = 6 or 7) with Diets I, II, and III containing 0, 36.7, and 73.5% rice bran and 0, 306, and 612 ppb As, respectively. Pigs were fed for 6 weeks, and their growth performance and daily activities were examined. Fecal, blood, and hair samples were collected immediately before and after the 6-weeks. At the end of the 6-weeks, pigs were slaughtered; the liver, kidney, muscle, and urine samples were collected. No pig showed any unhealthy signs throughout the trial. The average daily feed intake, average daily gain, and final body weight of Diet III pigs were lower (*p* ≤ 0.001) than Diet I pigs. The gain to feed ratios were not different among the treatments. The fecal, hair, kidney, and urinary As concentrations of both Diets II and III pigs were higher than Diet I pigs. The hair As concentration of Diet III pigs was higher than Diet II pigs, but no difference was found in the fecal, urinary, kidney, or muscle As concentrations between Diets II and III pigs. The blood and muscle As concentrations were below 10 ppb. These results suggest that 73.5% dietary rice bran inclusion compromised growth performance, whereas the 36.7% inclusion did not. The fecal As data imply that dietary As was poorly absorbed by the gastrointestinal tract. The tissue As data indicate that the absorbed As was rapidly cleared from the blood with some retained in various organs and others eliminated via urine. The hair As concentration was much higher than that of liver and kidney. The muscle As data suggest that the pork produced from the pigs fed a typical As-containing rice bran as used in this study is safe for human consumption.

## 1. Introduction

Arsenic (As), a semi-metal element in nature, is commonly found in water, soil, food, and other materials in both organic and inorganic forms [1,2]. Animals and humans can be exposed to As from the sources as drinking water and foodstuffs, such as seafood, mushrooms, and rice [2]. According to the International Agency for Research on Cancer (IARC), the inorganic form of As is a class-I non-threshold carcinogen to humans [3]. Consequently, chronic exposure to As from food sources can lead to cancer and numerous other diseases, such as diabetes, skin lesions, vascular disorders, and heart diseases [4,5,6].

Rice (*Oryza sativa* L.) is a staple food for nearly a half of the world’s human population [7,8]. The anaerobic growing conditions of flooded rice paddies and the unique physiology of the rice plant allow rice to take up As from water and soil in a very efficient manner, and then sequester it in different organs within the plant. As a result, the As content in rice is roughly 10 times higher than in other crops grown in the same region, even if the soil has limited anthropogenic contamination [9]. In the rice seed, the order of As concentrations is “rice hull (husk) > rice bran > milled rice grain” [10,11]. Rice bran—a rice production by-product—is frequently used as animal feed and human food ingredient due to its nutrition value [8]. Therefore, rice bran can be a potential source of As contamination in the chain of animal feed and human food.

Rice bran, as a feedstuff for agricultural animals, is rich in proteins (12 to 18%), lipids (14 to 22%), carbohydrates (50 to 80%, including 3 to 15% crude fiber), minerals, and vitamins [8,12,13]. Rice bran is incorporated into those practical diets for many animal species, including cattle, swine, and poultry [14,15]. Using rice bran as a feedstuff for meat-producing animals raises a critical question: whether or not the As in rice bran contaminates the meat or any other edible tissues of the animal following prolonged ingestion of the rice bran. Furthermore, the accumulation of residual As to a toxic level in the edible tissues of meat-producing animals would be considered not only a health concern for humans, but also an environmental problem [16]. Therefore, to avoid As poisoning through the foods of animal origin, it is crucial to understand the accumulation of residual As in different tissues of the animals fed with As-containing rice bran. Pork is the most consumed meat by the human population in the world [17]. Thus, the main objectives of this study are: (1) to evaluate the health status and growth performance of pigs fed with an As-containing rice bran, and (2) to investigate the retentional distribution of As among various pig tissues after a chronic exposure to the As-containing rice bran.

## 2. Materials and Methods

### 2.1. Selection of Rice Bran for the Study

We initially collected a total of six samples from six local rice mill plants representing six typical rice bran products in Mississippi and Arkansas in the year of 2018. The total As concentrations of these six samples were (mean ±SD, as-fed basis) 833 ± 25, 831 ± 32, 1030 ± 54, 958 ± 49, 884 ± 37, 484 ± 37 ppb, respectively. The sample with As at 884 ± 37 ppb from Arkansas, USA was subjectively chosen for this study, in which the total As was 833 ppb measured right before the start of this experiment. In terms of chemical forms, the inorganic As content was 87.99% including 74.28% arsenite (As^III^) and 13.71% arsenate (As^V^), and the content of other As forms was 12.01% that include 10.57% dimethylarsenic acid and 1.44% monomethylarsonic acid. We considered that this sample should well represent the typical rice bran products on the current market in the region.

### 2.2. Animals and Diets

All experimental protocols for caring, handling, and treatment of animals were approved by the Mississippi State University Institutional Animal Care and Use Committee. Twenty crossbred (Yorkshire × Landrace) growing gilts (initial body weight (BW) 26.3 ± 2.18 kg) were purchased from a local farm and transferred to an environment-controlled swine barn at Mississippi State University Leveck Animal Research Center. Gilts were individually penned and assigned to 3 dietary treatments according to a completely randomized experimental design (6 pigs in the control group and 7 pigs in each of the two treatment groups).

A corn and soybean meal-based control diet (the basal diet; Diet I) was formulated to meet or exceed the National Research Council (NRC, 2012) recommended requirements for energy and various essential nutrients (Table 1) [13]. Diet II and Diet III were formulated by inclusion of 36.7% (a moderate amount inclusion) and 73.5% (a high amount inclusion) As-containing rice bran into the basal diet, respectively. The contents of energy and essential amino acids in Diets II and III were balanced by adjusting the amount of corn and soybean meal used and supplementing poultry fat and various crystalline amino acids to meet the NRC (2012) recommended requirements. Table 1 presents the dietary ingredient composition (as-fed basis) and Table 2 presents the calculated and analyzed nutrient composition (as-fed basis) of the three experimental diets [13].

### 2.3. Animal Feeding Trial

After arrival from the local commercial farm, pigs were acclimated to the barn environment for a period of 10 days and then allowed ad libitum access to Diets I, II, or III according to the experiment design. Fresh water with no detectable As content (below the threshold level as set by the US government) was available to the pigs throughout the trial. All feeders and waterers were checked three times daily (0600 to 2200 h) to ensure the proper functions of the equipment. Orts were weighed or estimated (if less than 10 g) daily for feed intake calculation. Pigs’ BWs were measured at the beginning and the end of the feeding trial that lasted for 6 weeks. In addition, the pig’s feed intake was recorded throughout the feeding trial. The average daily gain (ADG), average daily feed intake (ADFI), and gain to feed (G:F) ratio were calculated for the overall 6-week feeding period accordingly.

Clinical observation was conducted daily with pigs’ activities being observed including eating behavior (appetite), drinking behavior, respiration behavior, standing posture, and walking behavior. Pigs’ hair coat appearance, fecal consistency, and laying-down posture were also observed daily [18].

### 2.4. Collection of Tissue Samples

Fresh fecal samples were collected at the beginning and also at the end of the 6-week feeding period, placed in 50-mL polypropylene centrifuge tubes individually, and stored at −20 °C in our laboratory. Hair samples were collected using a hair trimmer at the beginning and the end of the feeding trial, placed in separate envelopes, and stored at room temperature.

Before and after the 6-week feeding trial, blood samples (about 10 mL each) were also collected via jugular venipuncture with 10-mL vacutainers that were spray-coated with lithium heparin (Becton, Dickinson and Company, Franklin Lakes, NJ, USA). After collection, the blood samples were kept on ice for about 60 min before storage at −80 °C. All pigs were slaughtered in Mississippi State University Meat Science and Muscle Biology Laboratory, where the samples (about 20 to 30 g each) of liver, kidney, and semitendinosus skeletal muscle were collected. Fecal and urine samples were also collected at the slaughter in the laboratory. All the samples collected at the slaughter were kept on ice until being transferred to a −20 °C freezer for storage before sending out for total As determination.

### 2.5. Laboratory Arsenic Determination

The laboratory As determination was conducted by the Midwest Laboratories in Omaha, NE, USA, using a standard protocol of Method 6020 of the U.S. Environmental Protection Agency (https://www.epa.gov/esam). Briefly, the tissue and excreta samples were firstly digested using a combination of heat, nitric acid, and hydrogen peroxide, which destroys and solubilizes the tissue or excreta contents. Quality control of the sample preparation included a method blank, a laboratory control sample, and a duplicate of matrix spike. The sample digestates were then analyzed via an Inductively Coupled Mass Spectrometer (ICP-MS) operating under standard plasma conditions. The instrument calibration standards and verification checks were prepared from ISO Guide 34 standards purchased from separate vendors. The analytical quality control included an initial calibration verification, periodic continuing calibration verifications, and interference checks. The combination of sample preparation and analytical quality controls were implemented to support the validity and quality of the results.

### 2.6. Statistical Analyses

Animal growth performance data were analyzed with the General Linear Model procedure of SAS (version 9.3; SAS Institute Inc., Cary, NC) for ANOVA with diets as the main factor and pigs as experiment units. Means were separated with the PDIFF (adjust = T) option. Significance was set at *p* ≤ 0.05, and tendency was set at 0.05 < *p* ≤ 0.10.

To overcome various data limitations and ensure statistical robustness of the ANOVA, additional analyses were conducted employing two methods from the econometrics literature and using the software, Stata (StataCorp, College Station, TX, USA). In cases where all variables were measured with precision, the ordinary least squares regression model—a generalized form of the ANOVA model [19]—was used to include additional explanatory variables. Specifically, measures of baseline BW, baseline hair As concentrations, and baseline fecal As concentrations were included in the ordinary least squares regression model. Coefficients of interest were estimated using White (1980) heteroskedasticity-robust standard errors [20].

In cases where the dependent variable was imprecisely measured, the Tobit model [21] was used to estimate credible effects of diet on kidney and liver As concentrations, because some data were censored as “<10 ppb.” The Tobit model allowed for an agnostic analysis of the data without making arbitrary guesses about the true underlying values in cases when observations were censored [22]. In this study, censored data were an issue for the following dependent variables: fecal As concentrations (one left-censored observation), liver As concentrations (14 left-censored observations), and kidney As concentrations (4 left-censored observations). The application of Tobit model [21,22] solved this issue.

## 3. Results

### 3.1. Growth Performance and Behavior Observation

The effects of dietary inclusion of As-containing rice bran on growth performance of the pigs are presented in Table 3. There were no differences in the initial BW (*p* = 0.955) among the three dietary treatment groups. However, at the end of the feeding trial, the final BW of the pigs fed with Diet III were decreased by roughly 22.4% (*p* = 0.001) when compared to the pigs fed with Diet I; however, the difference in the final BW between Diet II and Diet I pigs was not significant (*p* = 0.313). Similarly, the ADG of Diet III pigs were decreased by roughly 36.7% (*p* < 0.001) when compared to Diet I pigs, while the difference in the ADG between Diet II and Diet I pigs was not significant (*p* = 0.204) either. The ADFI of Diet III pigs were also decreased by roughly 24.5% (*p* < 0.001) when compared to Diet I pigs, while the difference in the ADFI between Diet II and Diet I pigs was not significant (*p* = 0.215). The G:F ratios were not different among the three dietary treatment groups (*p* = 0.141).

Daily on-site observation showed that all pigs were of healthy looking and performed normal activities and behaviors. No sickness or poisoning symptoms and no diarrhea were observed on any pigs on any day, and no veterinarian visit was called for the pigs throughout the 6-week feeding trial.

### 3.2. Fecal Arsenic Concentration

**Figure 1:**
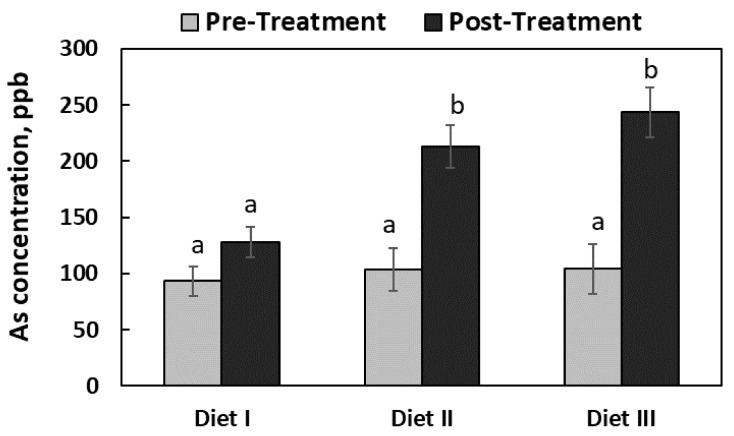
The concentrations of arsenic (As, as-is basis) in the feces of the pigs fed with three experimental diets. The rectangular bars represent the pattern of mean As concentrations in the feces before and after the feeding trial (pre- and post-treatment; *n* = 6 for Diet I, and *n* = 7 for Diets II and III). The error bars represent the pooled standard error of the means. The means labeled with different letters (a, b) differ (*p* < 0.05).

As shown in Figure 1, prior to the dietary treatment, there was no difference (*p* = 0.873) in fecal As concentrations (around 100 ppb) among the three groups of pigs. After the 6-week feeding trial, the fecal As concentration in Diet I pigs was 128 ± 26.4 ppb, which was not different (*p* > 0.100) from the concentration before the feeding trial. After the feeding trial, the As concentrations in Diets II and III pigs were increased (*p* = 0.003) to 213 ± 49.9 and 243 ± 54.3 ppb, respectively, and there was no difference (*p* = 0.975) in the As concentrations between the Diet II and Diet III pigs.

### 3.3. Hair Arsenic Concentration

**Figure 2:**
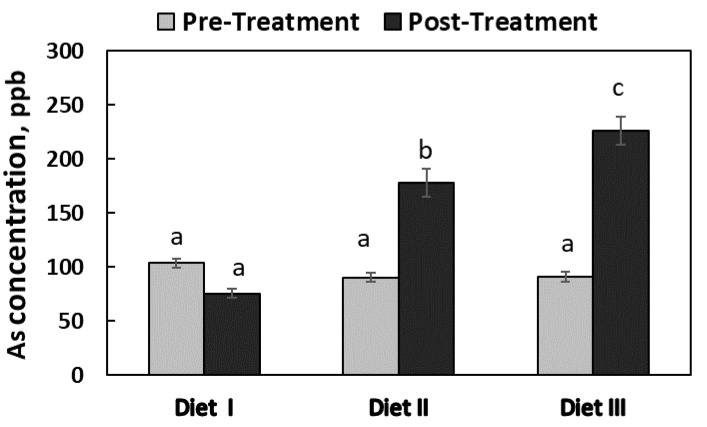
The concentrations of arsenic (As) in the hair of the pigs fed with three experimental diets. The rectangular bars represent the pattern of mean As concentrations in the hair before and after the feeding trial (pre- and post-treatment; *n* = 6 for Diet I, and *n* = 7 for Diets II and III). The error bars represent the pooled standard error of the means. The means labeled with different letters (a, b, c) differ (*p* < 0.05).

Before the 6-week feeding trial, the hair As concentrations were similar (*p* = 0.213) among the three groups of pigs, which ranged from 90 ± 8.5 to 104 ± 14.7 ppb (Figure 2). After the feeding trial, the hair As concentration of Diet I pigs (76 ± 9.9 ppb) was not increased (*p* < 0.100) at all, but the concentrations in Diet II (178 ± 31.5 ppb) and Diet III pigs (226 ± 32.5 ppb) were increased (*p* = 0.001) by roughly 98% and 148% from their pre-treatment concentrations, which were 90 and 91 ppb, respectively. In addition, the hair As concentration in Diet III pigs was higher than that of Diet II pigs by approximately 27% (*p* = 0.007) at the end of the feeding trial.

### 3.4. Urinary Arsenic Concentration

**Figure 3:**
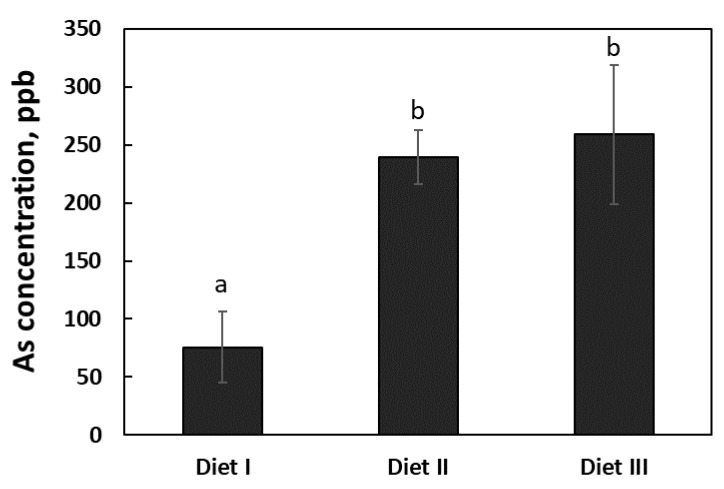
The concentrations of arsenic (As) in the urine of the pigs fed with three experimental diets. The rectangular bars represent the pattern of mean As concentrations in the urine after the feeding trial (*n* = 6 for Diet I, and *n* = 7 for Diets II and III). The error bars represent the pooled standard error of the means. The means labeled with different letters (a, b) differ (*p* < 0.05).

The urinary As concentrations of the pigs fed with Diets II and III for 6 weeks were 239 ± 61.0 ppb and 259 ± 158.5 ppb, respectively, which were approximately 220 and 245% higher (*p* = 0.016) than that (75 ± 30.2 ppb) of the pigs fed with Diet I (Figure 3). However, there was no difference (*p* = 0.746) in the urinary As concentrations between the pigs fed with Diets II and III.

### 3.5. Arsenic Concentrations of Internal Tissues

Before the feeding trial, no As was detected in the blood of any pigs tested (<10 ppb; data not shown). After the feeding trial, still no As was detected in the blood of these 20 pigs tested (Table 4). In terms of the skeletal muscle at the end of the feeding trial, the levels of As concentration in all the pigs were below the detectable threshold of 10 ppb (Table 4), so there was no difference between the rice bran feeding (Diets II and III) and the non-rice bran feeding (Diet I) groups. In terms of the liver at the end of the feeding trial, the As concentrations in the rice bran feeding groups (Diets II and III) were approximately 20 and 14 ppb, respectively, higher than that of the control group (Diet I). However, these differences were not statistically significant (*p* > 0.272). In terms of the kidney at the end of the feeding trial, the As concentrations in the rice bran feeding groups (Diets II and III) were approximately 21 and 18 ppb, respectively, higher (*p* < 0.001) than that of the non-rice bran feeding group (Diet I), and there was no difference (*p* = 0.249) between Diets II and III pigs.

## 4. Discussion

### 4.1. Effect of Arsenic-Containing Rice Bran on Growth Performance of Pigs

Previous research suggested that As is required by the pig as an ultra-trace mineral [23,24]. However, the level of As dietary requirement by the pig is uncertain [13], although As feed additives have been used in some swine farms, which is driven not only by some commercial benefits but also by certain traditional practices [1,18,25]. To humans, as aforementioned, inorganic As is a class-I non-threshold carcinogen and it can lead to numerous human diseases [6]. To the pig, evidence has indicated that upwards of 450 ppm arsanilic acid in the ration fed daily for one week could produce symptoms of acute poisoning [18]. In this study, however, all the pigs were healthy looking, performed normally, and displayed no poisoning symptoms throughout the trial.

Sun et al. (2008) reported that the total As content of rice bran products ranged from 0.71 to 1.98 mg/kg (or 710 to 1980 ppb) with the inorganic As ranging from 60 to 95% [11]. The total As content in the rice bran product used in this study was approximately 830 ppb, a mild level in general, and the total As contents in Diets II and III were approximately 450 and 630 ppb, respectively, which are far below the level of As in 450 ppm arsanilic acid as stated in Gary (1966) [18]. This is likely the reason why the pigs in this study did not show any clinical poisoning symptoms.

The growth performance data of this study clearly indicate that the ADFI and ADG of the pigs were negatively affected by the high level (approximately 70%) of dietary rice bran inclusion, but not significantly affected by the moderate level (approximately 35%) of the inclusion. Although the effect of dietary As content on the growth performance of pigs cannot be defined in this study, a conclusion regarding the effect of dietary rice bran as a whole on the ADFI and thus the ADG of pigs can be drawn. As shown in Table 2, both the crude fiber and contents in Diet III were higher than in Diet II, and the same was true for Diet II vs. Diet I. Same trends can also be found for the contents of crude fat, crude protein, gross energy, and ash (Table 2). Thus, the reduction of ADFI by dietary rice bran inclusion (at the level of 73% in this study) may be attributed to the contents of dietary fiber, crude fat, crude protein, gross energy, and ash. It is commonly recognized that fiber can stay in the digestive tract for a longer period and, therefore, reduces feed intake and weight gain. The negative effect of dietary fiber intake on energy and nutrient digestibilities is well documented in the literature [26,27,28,29,30], although Kaufmann et al. (2005) reported that the protein and amino acid digestibilities of rice bran have no relationship with the content of neutral detergent fiber [31].

Slightly different from the result of this study, de Campos et al. (2006) reported that the inclusion of 30% rice bran in a corn and soybean meal-based diet reduced ADFI by approximately 0.27 kg/day in growing-finishing pigs [32]. Casas et al. (2018) also reported that a dietary inclusion of full fat rice bran at 10 to 30% linearly reduced ADFI in finishing pigs. With weanling pigs [29], Warren and Farrell (1990) even reported that the ADFI was slightly increased when a defatted Australian rice bran was included from 10 to 30% [33]. The discrepancies between this present study and these afore-mentioned reports may be attributed to the differences in the chemical compositions of the neutral detergent fiber and crude fat in different rice bran products, as well as the different pigs, used in the studies.

As shown in Table 3, the G:F ratio was not significantly affected by the dietary inclusion of rice bran, which suggests that the reduced final BW of Diet III pigs was mainly due to the high level of dietary fiber inclusion and the reduced ADFI. These results are partially in agreement with Casas et al. (2018), who reported that the G:F ratio and final BW of pigs were not affected by the inclusion of rice bran [29]. Several factors, such as the dietary nutrient ratio balance and the health status of pigs, can affect the G:F ratio. The contents of dietary net energy, essential amino acids, as well as minerals and vitamins, are similar among the three experimental diets (Table 2). In addition, no pig showed any unhealthy activities during the entire feeding period. Thus, the feed efficiency was not significantly affected by the dietary inclusion of rice bran at a level up to 73%.

### 4.2. Transportation and Distribution of Arsenic in Pig Body

To date, the studies of As nutrition in pigs are not available or are very limited, so data regarding the As absorption, transportation, distribution, retention, and excretion, in and by the pig are scarce. Rice bran, a widely used animal feed, usually contains a high amount of As [11]. Thus, this present study was designed to investigate the tissue distribution of As (released from the rice bran consumed) within the pig body, and the fecal and urinary excretion of As out of the pig body.

It has been known that the As component from dietary source is readily absorbed in the intestine and rapidly transported by the blood stream throughout the body [34]. In pigs approximately 25% of dietary As are usually absorbed, whereas in humans the absorption rate is much higher, approximately 80% [35,36]. After absorption, most of the As is cleared rapidly from the blood stream of pigs with some being retained in various organs and others being eliminated through feces and urine [25]. In this study, the concentrations of As in the blood of the pigs were below the detection level, which, however, is discrepant from the results of Ledet et al. (1973), who reported that the average As concentration in the blood of pigs was 1.80 ppm during the 27-day feeding period [25]. The discrepancy between Ledet et al. (1973) and ours might be due to the low dietary As concentrations in our study, which were 448 and 633 ppb (analyzed values) for Diets II and III, respectively, whereas the pigs of Ledet et al. (1973) were fed the diet contained 1000 ppm arsanilic acid [25]. Thus, it can be predicted that the concentration of As in pig’s blood will be very low and, in most cases, undetectable when a mild amount of As (roughly 400 to 600 ppb) is fed.

To study the retentional distribution of As in various tissues, this study measured the total As concentrations in the liver, kidney, muscle, and hair samples of the pigs fed with or without the As-containing rice bran. The results showed that the As retention is less than or around 20 to 30 ppb in the liver and kidney of the pigs fed the moderate to high level of As-containing rice bran (Table 4). The As concentration in the muscle was under detection limit (<10 ppb) of the updated detection method current available for analytical laboratories. Similar As distribution pattern was observed by Ledet et al. (1973), who reported that the concentrations of As in the liver and kidney were much higher than in the muscle of the pigs consumed high As diets [25,37]. Ledet et al. (1973) reported that the As concentrations in liver and kidney were close (9.67 and 8.33 ppm, respectively) in the pigs fed with 1000 ppm As-containing diet. Moreover, López-Alonso et al. (2007) investigated the concentrations of As and some other essential metal elements in muscle, liver, and kidney of pigs in Galicia, Spain [38]. The authors reported that appreciable As contents were found in the liver and kidney, whereas As was not detected in most (98%) muscle samples.

The major biochemical mechanism of As retention in body tissues is that the arsenicals, which include trivalent, monomethyl, and dimethyl As that have high affinity for sulfhydryl groups, can bind to the reduced cysteine residues in peptides or proteins [39]. By this mechanism, As can be distributed ubiquitously to all major tissues; however, the actual capacity of As retention may vary in different tissues. For example, Ducoff and Neal (1948) used [^76^As] sodium arsenite to study As tissue distribution patterns and excretion rates in rats and rabbits [38]. Rats retained the most of As in their red blood cells, with smaller concentrations in the spleen, heart, lungs, kidneys, and liver. On the other hand, the As was lowest in the blood and highest in liver, kidneys, and lungs in rabbits [40,41]. The As tissue distribution pattern observed in this present study is principally supported by those previous studies in pigs, rats, and rabbits.

Arsenic retention was the highest in the hair when compared to the other tissues tested in this study, whereas no hair As concentration data for pigs were found in the literature. Olguín et al. (1983) studied a human population exposed with As and found that the second highest As deposition was in the hair (1240 ± 610 ppb) followed by the nails (4550 ± 3250 ppb) [42]. In addition, Katz (2019) summarized the hair As concentration data from multiple studies and claimed that the hair As concentration is positively correlated with and can be considered as a biomarker for evaluation of As toxicity in humans [43]. The scientific basis of this claim is that the keratin in hair is rich in disulfides that can easily incorporate As into the growing portion of hair root [43]. These references [42,43] support our findings in this study that the pigs’ hair retained the highest level of residual As after chronic exposure to dietary As.

### 4.3. Excretion of Arsenic from Pig Body

Along with the tissue distribution pattern, the concentrations of As in the feces and urine of the pigs were also measured in this study to explore the pattern of As excretion from pig body. The results of this study concerning the fecal As content provide some novel data on the pattern of As excretion. As illustrated in Figure 2, the fecal As concentrations were similar among the three groups of pigs before the feeding trial. However, after the 6-week feeding trial, the fecal As concentrations were increased drastically in pigs fed Diets II and III, which suggest that the degree of gastrointestinal As absorption was not high. This finding agrees with Mandal (2017) who stated that the As compounds are poorly absorbed in the intestine of pigs (only about 30% of dietary intake) when compared to rodents and humans (approximately 90% of the dietary intake) [44]. As a result, most of the unabsorbed As was excreted through the feces of the pigs. That being said, please also keep in mind that since As can circulate in the enterohepatic and can be reabsorbed by the intestine, an increased fecal As excretion may not necessarily be consistent with a poor As absorption.

The fact that As was excreted in the urine is an evidence of As absorption. In terms of As excretion through urine, studies with laboratory animal models have shown that the inorganic As is metabolized by the reduction of arsenate (As^V^) to arsenite (As^III^), followed by the sequential methylation to monomethylarsonic acid (MMA) and dimethyl As acid (DMA). These methylation reactions have traditionally been regarded as a detoxification mechanism since the methylated metabolites exert less reactivity and acute toxicity with tissue constituents than the inorganic As [34,45,46,47]. The methylated forms of As are water soluble and mostly can be eliminated from the body via glomerular filtration of the kidney or through urinary excretion [48,49,50]. Most mammalian species can efficiently eliminate As from the body after withdrawal of the As-containing diet [37].

In this study, the As concentration was increased by more than 200% in the urine of pigs fed Diets II and III relative to Diet I. There is a lack of reference value in the literature to support this finding of ours regarding urinary As excretion pattern in pigs. Previous studies in humans and experimental animals suggested that urination is one of the primary routes for the arsenical compounds to be eliminated from the body, and the intake of As from dietary or other sources affects the urinary As concentration [49,51,52]. Bae et al. (2013) studied the relationship between daily dietary As intake and the urinary As concentration in a Korean population and found a significantly positive (r = 0.096, *p* < 0.05) relation between the two parameters, which suggested that the dietary As intake affects the total urinary As concentration [53]. A study by Choi et al. (2012) also found high As concentrations in the urine of seafood-consuming population in Korea [54]. Therefore, the high As concentration in the urine of pigs fed high As diets suggests that pigs are capable of efficiently eliminating most of the absorbed dietary As through urine.

### 4.4. Human Health Implication

Inorganic As is a class-I non-threshold carcinogen to humans, and a chronic exposure to As from food sources can also lead humans to many other diseases [4,5,6]. Thus, it is imperative to evaluate the As contamination in the food chain for humans. Pig is considered as an excellent, if not the best, animal model for studying human nutrition and health [55]. The results obtained from this study have at least two implications for human nutrition and health. First, the As tissue distribution data may shed some light for human nutritionists and toxicologists to understand the As tissue distribution and toxicity in humans, considering that rice is a staple food for over half of the world’s population and the accumulation of As by rice from the environment is so efficient [56,57]. Secondly, the result of As concentration in skeletal muscle of the pigs suggests that the pork (with the major component being skeletal muscle) produced from the pigs fed with rice bran that contains a typical mild level of As (around 800 ppb) should be safe for humans to eat. According to Saifullah et al. (2018), an acute minimal lethal dose of As in adults is estimated to be 1 mg/day per kilogram of BW [58].

## 5. Conclusions

Overall, the inclusion of a moderate level of rice bran in the diet did not significantly influence the growth performance of pigs; however, the growth performance was significantly compromised with the inclusion of a high level of rice bran. The fecal As content was increased in pigs fed the As-containing rice bran diets, which implies that the dietary As might be poorly absorbed in the gastrointestinal tract of pigs. The results of As tissue distribution indicate that the absorbed As was rapidly cleared from the blood stream and deposited in various tissues. A high amount of As was appeared in the hair of the pigs, while limited amounts of As were retained in the kidney and liver (about 20 to 30 ppb). The As concentration in skeletal muscle of the pigs was negligible (<10 ppb). The increased urine As concentration suggests that pigs may be able to efficiently eliminate non-retained As from the body. Although the rapid elimination of As from the pigs’ body may raise an environmental pollution concern, the pork resulting from the pig fed with rice bran that contains a typical mild level of As should be safe for human consumption.

## Figures and Tables

**Table 1:** Composition (%, as-fed basis) of the three experimental diets formulated for the growing pigs.

Item	Diet ^1^		
	Diet I	Diet II	Diet III
*Ingredient*			
Corn	78.475	43.958	0.000
Rice bran ^2^	0.000	36.732	73.464
Soybean meal	18.400	14.400	18.500
Poultry fat	0.000	2.100	6.000
L-Lysine●HCl	0.450	0.480	0.290
DL-Methionine	0.060	0.065	0.050
L-Threonine	0.120	0.170	0.100
L-Tryptophan	0.030	0.030	0.001
L-Isoleucine	0.000	0.040	0.000
L-Valine	0.040	0.070	0.000
L-Cysteine●HCl, anhydrous	0.060	0.090	0.080
Limestone	0.850	1.200	1.200
Dicalcium phosphate	1.200	0.350	0.000
Salt	0.180	0.180	0.180
Mineral premix ^3^	0.070	0.070	0.070
Vitamin premix ^3^	0.065	0.065	0.065
*Total*	100.000	100.000	100.000

^1^ Diet I = the control diet; Diet II = the moderate rice bran diet; Diet III = the high rice bran diet. ^2^ The analyzed arsenic concentration in the rice bran used was 833 ppb. ^3^ Both swine trace mineral premix (NB-8557D) and swine vitamin premix (NB-A16508A0) were donated from Nutra Blend, LLC (Neosho, MO) that provided (per kilogram of diet): S, 0.06 g; Cu, 7.7 mg; Fe, 51.5 mg; I, 0.14 mg; Mn, 15.5 mg; Zn, 51.4 mg, Se, 0.14 mg; vitamin A, 2293 IU; vitamin D3, 573 IU; vitamin E, 11.5 IU; vitamin B2, 2.15 mg; niacin, 21.5 mg; vitamin B5, 7.16 mg; and vitamin B12, 10.0 µg.

**Table 2:** Calculated and analyzed nutrient composition (%, or as indicated) of the three experimental diets (as-fed basis).

Item	Diet ^1^		
	Diet I	Diet II	Diet III
*Calculated composition*			
Net energy, kcal/kg	2518	2508	2528
Total crude protein	15.7	15.6	18.7
SID ^2^ crude protein	13.2	8.0	9.5
SID lysine	0.98	0.98	0.99
SID methionine + cystine	0.56	0.55	0.56
SID threonine	0.59	0.59	0.59
SID tryptophan	0.17	0.17	0.17
SID arginine	0.88	0.94	1.23
SID histidine	0.36	0.34	0.40
SID leucine	1.24	1.03	0.99
SID isoleucine	0.53	0.51	0.54
SID valine	0.64	0.64	0.66
SID phenylalanine + tyrosine	1.05	0.97	1.12
Linoleic Acid	1.62	2.05	2.69
Crude fiber	2.22	4.19	6.24
Neutral detergent fiber	9.76	15.43	20.69
Acid detergent fiber	3.20	6.41	9.83
Total Ca	0.66	0.66	0.66
STTD ^3^ P	0.35	0.33	0.43
Arsenic, ppb	0.00	306	612
*Analyzed composition* ^4^			
Dry matter	88.2	90.3	92.2
Gross energy, kcal/kg	3957	4729	5227
Crude protein	15.1	15.5	18.2
Crude fat	1.8	10.2	18.2
Crude fiber	3.16	3.36	4.36
Ash	4.83	6.99	9.46
Arsenic, ppb	134	448	633

^1^ Diet I = the control diet; Diet II = the moderate rice bran diet; Diet III = the high rice bran diet. ^2^ SID = standardized ileal digestible. ^3^ STTD = Standardized total tract digestible. ^4^ Feed proximate and energy analyses were conducted by the Essig Animal Nutrition Laboratory (Mississippi State, MS). Feed arsenic concentrations were analyzed by the Midwest Laboratories (Omaha, NE).

**Table 3:** The growth performance of the pigs fed with three experimental diets ^1^.

Item ^2^	Diet ^3^			*p*-Value ^4^
	Diet I	Diet II	Diet III	
Initial BW, kg	26.2 ± 2.52	26.2 ± 1.96	26.5 ± 2.07	0.955
Final BW, kg	72.0 ^a^ ± 5.50	68.0 ^a^ ± 5.33	55.9 ^b^ ± 8.81	0.001
ADG, kg/day	1.09 ^a^ ± 0.08	0.99 ^a^ ± 0.09	0.69 ^b^ ± 0.18	<0.0001
ADFI, kg/day	2.29 ^a^ ± 0.23	2.14 ^a^ ± 0.17	1.73 ^b^ ± 0.23	<0.0001
G:F ratio ^5^	0.47 ± 0.02	0.46 ± 0.02	0.40 ± 0.10	0.141

^1^ Each value is a mean ± standard deviation (*n* = 6 for Diet I and *n* = 7 for Diets II and III). ^2^ ADG = average daily gain; ADFI = average daily feed intake; G:F ratio = gain to feed ratio. ^3^ Diet I = the control diet; Diet II = the moderate rice bran diet; Diet III = the high rice bran diet. ^4^ *p*-value was obtained from ANOVA test. ^5^ A borderline statistical significance (0.05 < *p* < 0.10) was obtained for the G:F ratios between Diet III and Diet I pigs when the ordinary least squares regression model was used. ^a,b^ Means within a row that have different superscripts differ (*p* < 0.05).

**Table 4:** The arsenic (As) concentrations (ppb, as is) in different tissues of the pigs fed with three experimental diets ^1^.

Tissue	Diet ^2^			F-Stat ^3^	*p*-Value ^4^		
	Diet I	Diet II	Diet III		II vs. I	III vs. I	III vs. II
Blood	<10	<10	<10	NA	NA	NA	NA
Muscle	<10	<10	<10	NA	NA	NA	NA
Liver	<10	19.6	13.6	0.16	0.296	0.272	0.696
Kidney	<10 ^a^	21.0 ^b^	18.0 ^b^	1.40	<0.001	<0.001	0.249

^1^ The values above 10 ppb represent the comparison values instead of the actual As concentrations, indicating how much more As present in the tissues of Diet II or III pigs than that of Diet I pigs. The detection limit of the method used in the analytical laboratory was 10 ppb. ^2^ Diet I = the control diet; Diet II = the moderate rice bran diet; Diet III = the high rice bran diet. ^3^ F-stat = the F statistic shown only for the hypothesis test of Diet III vs. Diet II. ^4^ *p*-values correspond to the hypothesis tests of Diet II vs. Diet I, Diet III vs. Diet I, and Diet III vs. Diet II, respectively, using Tobit model. ^a,b^ Means within a row that have different superscripts differ (*p* < 0.05).

## Abbreviations

As	Arsenic
ADG	average daily gain
ADFI	average daily feed intake
BW	body weight
G:F	gain to feed ratio
IARC	International Agency for Research on Cancer
ICP-MS	Inductively Coupled Plasma—Mass Spectrometry
NRC	National Research Council
PDIFF	*p*-values for differences
ppb	parts per billion
ppm	parts per million
